# Retrospective Analysis of the Impact of a Dietitian and the Canadian Nutrition Screening Tool in a Geriatric Oncology Clinic

**DOI:** 10.3390/nu17091591

**Published:** 2025-05-06

**Authors:** Harriet Ho, Linda Cerullo, Rana Jin, Susie Monginot, Shabbir M. H. Alibhai

**Affiliations:** 1 Department of Medicine, University Health Network, 200 Elizabeth Street Room EN14-214, Toronto, ON M5G 2C4, Canada; CheukHeiHarriet.Ho@uhn.ca; 2 Department of Supportive Care, Princess Margaret Cancer Centre, University Health Network, 610 University Avenue, Toronto, ON M5G 2M9, Canada; 3 Department of Medicine, Institute of Health Policy, Management, and Evaluation, and Institute of Medical Sciences, University of Toronto, 6 Queens Park Crescent West 3rd Floor, Toronto, ON M5S 3H2, Canada

**Keywords:** geriatric assessment, malnutrition, nutrition assessment, geriatric oncology, Canadian nutrition screening tool

## Abstract

Introduction: Canada’s aging population is leading to an increased number of older adults being diagnosed with cancer. This population faces unique challenges, including frailty, comorbidities, polypharmacy, and malnutrition, which can negatively affect treatment outcomes. The role of registered dietitians (RDs) in managing nutrition-related issues in this population is well-documented, but there is limited research on their integration into geriatric oncology clinics. We evaluated the impact of integrating a registered dietitian (RD) into the Older Adult with Cancer Clinic (OACC) at the Princess Margaret Cancer Centre, Toronto, Canada. Materials and Methods: A retrospective chart review was conducted of older adult cancer patients seen at the OACC, comparing outcomes before and after the RD’s integration. The focus was on weight characteristics and change, malnutrition screening/identification, and management. The two-item Canadian Nutrition Screening Tool (CNST) was introduced during the RD’s integration and was also examined to see its usefulness in identifying malnutrition risk. Chi-squared tests and t-tests were used for data analysis. Results: The pre-cohort (n = 140) had a mean age of 80.2 years, 48.6% female, and 77.9% vulnerable (Vulnerable Elders Survey (VES-13) ≥ 3). The post-cohort (n = 117) had a mean age of 81.4 years, 59.8% female, and 80.3% vulnerable (VES-13 ≥ 3). Weight change within 3 ± 1 months after the initial OACC consult was similar between pre and post groups with −1.4 kg and −1.2 kg, respectively (*p* = 0.77). Patients at nutritional risk, as determined by the OACC team, generated significantly more referrals to the RD in the post group (100% vs. 36.4%, *p* < *0*.001). Among patients who had CNST screening and saw the RD, there was a higher rate of high nutrition risk among CNST-positive compared to CNST-negative patients (67.2% versus 44.4%, respectively). After the integration of the RD, a greater number of patients at nutritional risk received nutritional education and referrals to other healthcare professionals (43 versus 1). Conclusions: The integration of an RD into the OACC led to improved referral rates, nutritional education, and referrals to other healthcare professionals. Moreover, patients who were CNST positive were more likely to have high nutritional risk.

## 1. Background

The majority of cancer diagnoses and deaths occur in older adults aged 65 and older, and, with Canada’s older adult population expected to double in the next 20 years, the number of older Canadians with cancer will also increase [1]. Older adults with cancer face greater complexity compared to younger adults due to comorbidities, polypharmacy, and functional and cognitive impairments, making them more vulnerable to treatment toxicity and non-adherence [2]. The comprehensive geriatric assessment (CGA), considered the gold standard for managing older adults with cancer, has been shown to reduce treatment toxicity, improve quality of life, and enhance patient-centred care [3,4,5,6].

Nutrition is particularly crucial in this population, as age-related challenges such as cachexia (loss of lean body mass and fat) and sarcopenia (loss of muscle mass) are often exacerbated by malnutrition, leading to frailty and reduced quality of life [7]. Malnutrition, defined as the insufficient acquisition of nutrients, is highly prevalent in older adults with cancer, especially those with head and neck or gastrointestinal cancers, where digestion and appetite are impacted [8]. Nearly 66% of older adults are malnourished, and a cancer diagnosis increases the risk of malnutrition 14-fold [8]. Malnutrition has been linked to higher chemotherapy toxicity and poorer survival outcomes, making malnutrition an area of concern [9].

Current research strengthens the benefits of nutritional support by a registered dietitian (RD) in improving nutrient intake, quality of life, and physical function in cancer patients, with randomized controlled trials (RCTs) showing positive effects on survival, chemotherapy compliance, and weight maintenance [10,11,12,13]. However, access to RDs is limited, with current RD-to-patient ratios reaching up to 1:2300 compared to the ideal 1:120, leaving many patients without support and facing conflicting advice [14,15]. Successful RD interventions highlight the importance of early screening, early referrals, and ongoing education, but data on integrating RDs into geriatric oncology clinics remain limited [11,15,16].

At the Princess Margaret Cancer Centre’s (PM) Older Adults with Cancer Clinic (OACC), a study by Abu Helal et al. in 2022 found that 44.2% of patients seen between June 2015 and June 2018 were at nutritional risk, yet 39.8% of these patients had no recommendations addressing this risk, illustrating how malnutrition is often not adequately managed, even in highly specialized settings [17]. As the role of an RD has been shown to help prevent malnutrition as well as improve patient nutritional care, in September 2022, the OACC introduced a 0.2 full-time equivalent (i.e., one day a week) RD to help address nutritional issues. Simultaneously, the Canadian Nutrition Screening Tool (CNST) was introduced to help screen for malnutrition risk and guide referrals to an RD. The CNST is a 2-item yes/no questionnaire that examines whether there was any weight loss in the past six months and eating less than usual in the last week; ‘yes’ to both items indicates a positive CNST, which indicates a risk for malnutrition, prompting a referral to an RD [18]. Additionally, if the CNST is negative but the patient is deemed at risk for malnutrition from clinical judgement by the OACC clinical nurse specialist and geriatrician assessment, then a referral to an RD is made. Other validated and recommended nutrition screening tools, such as the Malnutrition Screening Tool (MST), the Mini-Nutritional Assessment Short Form Revised (MNA-SF), the Nutrition Risk Screening (NRS-2002), and the Malnutrition Universal Screening Tool (MUST), were considered. However, each of these requires a longer time to administer in comparison to the CNST [19]. The CNST was also introduced in the inpatient setting in 2022 at PM based on the Canadian malnutrition task force review and recommendations [20]. Direct comparisons between instruments were lacking. Of note, the validation of the CNST was performed in an inpatient setting and was used in an outpatient population with inflammatory bowel disease in two centers, and its effectiveness in older adults with cancer in an outpatient setting remains untested [18,21].

Taking into account the integration of both a RD and the CNST at the OACC, the objectives of this study were (1) to assess nutrition-related patient outcomes prior to and after the integration of a dietitian in the OACC, (2) to assess whether malnutrition was adequately addressed when identified by the dietitian, and (3) to examine the usefulness of the CNST in identifying malnutrition risk.

## 2. Materials and Methods

**Figure 1:**
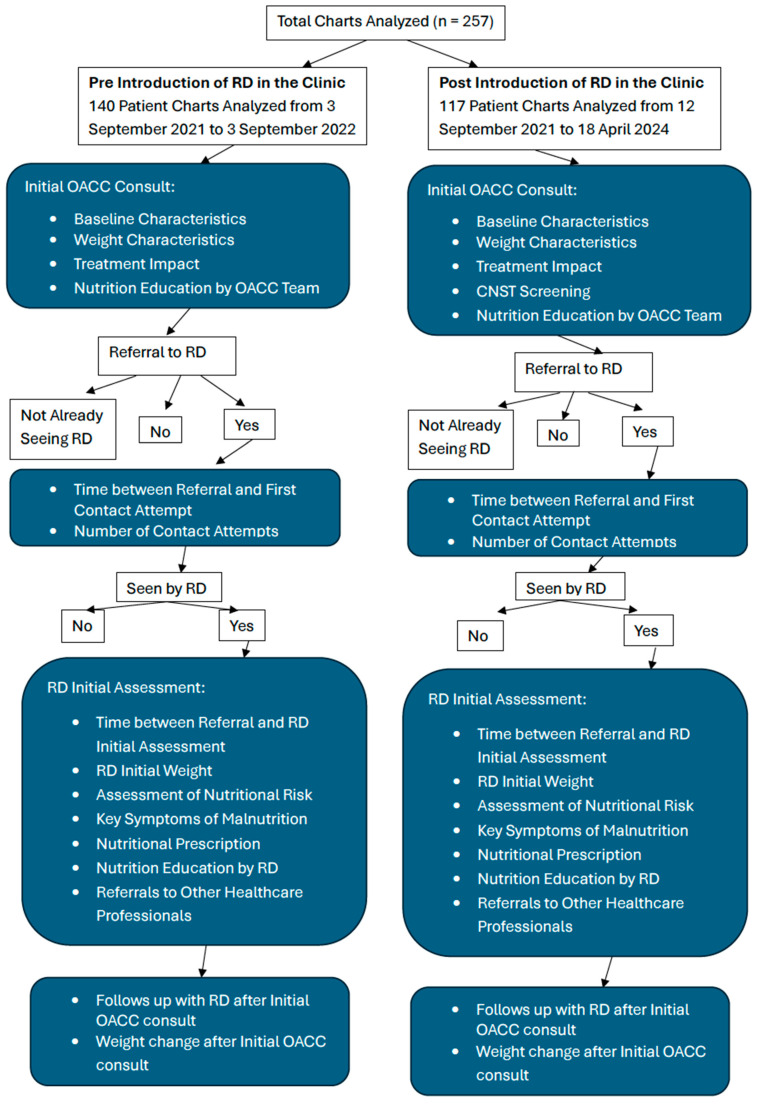
Schematic diagram of the study design, workflow, and key outcome measures. Note: “OACC” = Older Adult Cancer Clinic, “RD” = Registered Dietitian, “CNST” = Canadian Nutrition Screening Tool.

We conducted a retrospective chart review of the OACC database with a before–after design to assess nutrition-related patient outcomes when an RD was integrated into the OACC from 12 September 2022 to 18 April 2024, compared to the period immediately before the RD was integrated from 3 September 2021 to 18 August 2022. The gap between 18 August 2022 and 12 September 2022 represents a transition period where the RD was formally introduced, allowing time for organizational alignment and integration of the RD into the OACC’s routine operations. In the pre-RD group, patients received usual care, which included assessments conducted by OACC physicians and nurses who evaluated patients’ weight loss, blood test results, and overall nutritional risk (malnourished, at risk, or normal). Based on these assessments, patients were advised or referred to community or PM-based RDs for follow-up [16]. Usual RD care at PM primarily focused on patients who are currently receiving or were up to three months post-cancer-directed therapy. In general, diet prescription is based on clinical judgement and patient symptoms while considering where patients are in their treatment trajectory, e.g., pre-treatment or receiving active systemic therapy. For example, the use of a low-fiber diet for patients with ovarian cancer is recommended because of the increased risk of bowel obstruction” [22]. Common nutrition interventions include high-calorie high high-protein dietary modifications as well as managing symptoms such as anorexia, vomiting, dysgeusia, mucositis, constipation, and/or diarrhea. These recommendations are usually aimed at patients and families. Healthcare professionals may also use these recommendations to assist in enhancing the patient’s overall nutritional status [23]. The CNST was not part of usual care during this period. A schematic diagram detailing the study design, workflow, and key outcome measures can be seen in Figure 1.

This study included consecutive older adult patients (age 65 or older) seen at the OACC at different stages of the cancer journey (i.e., pretreatment, on active treatment, or post-treatment). Demographic variables were obtained from the OACC clinical database and included age, sex, Vulnerable Elders Survey (VES-13) score [24], disease site, treatment intent, reason for referral, current treatment, proposed treatment, and the eight domains of the CGA [11,14]. At the OACC, a CGA is conducted by a clinical nurse specialist and geriatrician for all new patients. The eight domains follow the American Society for Clinical Oncology’s (ASCO) geriatric oncology updated guidelines (2023) and include comorbidities, functional status, falls risk, medication optimization, social support, mood, cognition, and nutrition, as previously described [3,25].

Nutrition was classified as normal (i.e., low nutritional risk), at risk, or malnourished by the OACC MD. Low-risk patients would have a BMI between 22 and 27 kg/m^2^, stable weight, adequate dietary intake, no physical signs of nutrient deficiencies, and bloodwork within normal ranges. At-risk patients may have a BMI below 22 kg/m^2^ (underweight) or above 27 kg/m^2^ (overweight/obese), with unintentional weight loss of 3–4.9% over the past 6 months, inconsistent nutrient intake, or mild signs of nutrient deficiencies. Malnourished patients typically presented with a BMI below 22 kg/m^2^ or severe weight loss exceeding 5–10% in 6 months, along with inadequate nutrient intake, visible signs of malnutrition (e.g., muscle wasting), and abnormal laboratory values.

### 2.1. Outcome Measures

Key outcomes for each objective include (1) weight characteristics (prior weight loss, and weight change 3 months after OACC consultation; (2) malnutrition screening/identification (time from referral to contact attempt and initial assessment, referrals to RD, RD nutritional risk judgement), malnutrition assessment and management (key symptoms of malnutrition, nutritional prescription, nutrition education, referrals to other healthcare specialists); (3) CNST screening result and comparison with OACC MD and RD nutritional risk judgement. An exploratory outcome was the impact of nutritional risk on treatment recommendation by the OACC and on the final treatment plan. Full details of the outcome measures are shown in Table 1.

### 2.2. Data Extraction

Data were extracted by HH using a customized Excel spreadsheet. To ensure data accuracy, a double data abstraction process was conducted for all patients. Any discrepancies or missing data were reviewed and clarified in consultation with LC and SA.

### 2.3. Sample Size

The sample size calculation was performed using an online statistical tool (ClinCalc) for a two-independent-sample study design with a dichotomous endpoint with an alpha (Type I error rate) of 0.05, a power of 0.8, and an enrolment ratio of 1:1. Based on these parameters, the required sample size was determined to be 41 charts per group (pre and post) to detect a minimum 30% increase in the proportion of patients receiving nutritional assessments (from 25% to 55%) for a total of 82 charts. This rate is based on the study by Abu Helal et al., which reported a rate of 25% [17]. To ensure the precision of the study findings, the confidence interval was also considered. With a sample size of 100 charts in the pre-RD period, the estimate would be within ±9% of the true proportion, assuming a baseline rate of 30% for comprehensive nutrition assessments. Therefore, a total of 200 charts (100 in the pre-RD period and 100 in the post-RD period) were selected as a minimum that would be sufficient to detect a meaningful improvement in the quality of nutritional assessments, as well as providing sufficient precision for other estimates.

### 2.4. Statistical Analyses

All data analyses were conducted using Microsoft Excel 2016 (Microsoft Corp, Redmond, WCA, 98052). Baseline characteristics and outcomes were described using means or medians for continuous variables and counts for categorical variables. *T*-tests, Wilcoxon Rank sum tests, and chi-squared tests were used to compare means, medians, and counts, respectively, between the pre and post group. Patients were further stratified into at-risk or malnourished groups based on the OACC MD nutritional risk judgment to compare referral rates to the RD and nutrition education received.

## 3. Results

A total of 257 charts were reviewed, 140 in the pre-period and 117 in the post-period. The baseline characteristics of patients in the full cohort and pre and post groups are summarized in Table 2. The pre and post populations had mean ages of 80.2 and 81.4 years and were 48.6% and 59.8% women, respectively. Of the pre and post populations, respectively, 77.9% and 80.3% had VES-13 scores of 3 or higher, suggesting increased vulnerability or frailty [20]. The three most common disease sites amongst patients seen in the pre population were head and neck (29.3%), gastrointestinal (24.3%), and gynecological (13.6%). In the post population, it was gynecological (18.8%), head and neck (17.1%), and gastrointestinal (16.2%). About half the patients had a curative treatment intent (52.9% and 45.3% in the pre and post population, respectively). The majority of patients were referred in the pre-treatment setting, with 80.0% and 73.5% for the pre and post population, respectively. From the nutrition domain, 15.0% and 35.0% were malnourished, 85.0% and 53.8% were at risk for malnutrition, and 0% and 11.1% were considered low (normal) nutritional risk in the pre and post population, respectively. Additional baseline characteristics are shown in Table 2.

### 3.1. Objective 1: Nutrition-Related Patient Outcomes

#### 3.1.1. Weight Characteristics at Initial OACC Visit, Initial RD Assessment, % Weight Change at RD Assessment, Prior Weight Loss, Weight Change After OACC Visit

The pre and post population had a mean body mass index of 25.1 kg/m^2^ and 24.5 kg/m^2^, respectively (Table 3). Prior weight loss was very common, with 84.4% of patients self-reporting some type of weight loss prior to the OACC visit. The median quantifiable weight loss was 6.8 kg and 6.0 kg for the pre and post groups over a median time frame of 6 months, respectively (*p* = 0.19, Table 2). From the self-reported weight loss, 41/102 (40.2%) in the pre group and 39/89 (43.8%) patients in the post group were classified as having clinically significant weight loss according to the University Health Network Clinical Nutrition Prioritization Matrix (Supplementary Figure S1) (*p* = 0.61) [26]. The pre group had a mean weight change over 90 days of −1.4 kg after the OACC consult, while the post group had similar weight changes (−1.2 kg), with no statistical difference (*p* = 0.77). Of the patients who had a reportable weight change, weight loss of 5 kg or more was observed slightly more in the pre group (18.6%) than in the post group (14.8%), *p* = 0.52.

**Figure 2:**
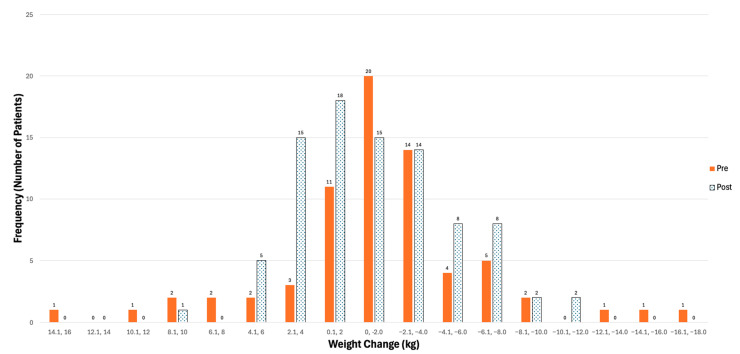
Distribution of weight changes (kg) (follow-up weight—initial weight) 3 months after OACC visit in pre and post patients. Note: “OACC” = Older Adult with Cancer Clinic, “kg” = kilograms.

Figure 2 demonstrates that the most common weight changes for both pre and post groups were between 0 and −2.0 kg.

#### 3.1.2. Treatment Impact

The proportion of patients for whom nutrition was mentioned as influencing the OACC treatment recommendation significantly increased post-intervention (22.2%) compared to pre-intervention (5.7%) (*p* < 0.001). However, there was no significant difference in the inclusion of nutrition concerns by the referring oncologist in the final treatment plan between the two periods (1.4% pre vs. 0.9% post, *p* = 0.93) (Supplementary Table S1).

### 3.2. Objective 2: Malnutrition Screening/Identification

#### 3.2.1. Timeliness of Assessment, Referral to First Contact Attempt, Number of Contact Attempts, Referral to Initial RD Assessment

The median time from referral to first contact attempt by the RD was 14 days for both the pre and post group (Supplementary Figure S2). The median time between referral and initial RD assessment was 14 days and 21 days for the pre and post groups, respectively (Table 4).

#### 3.2.2. Referrals to Registered Dietitian

**Figure 3:**
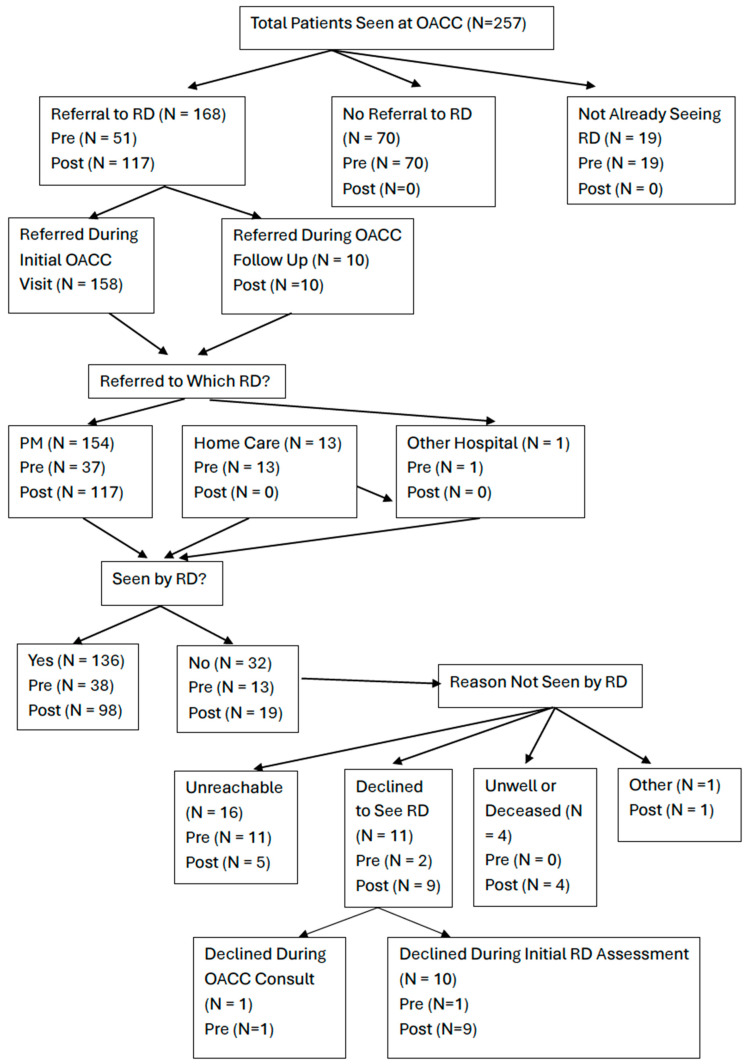
Flowchart illustrating the referral to the RD process and whether patients were seen by the RD. Note: “OACC” = Older Adult with Cancer Clinic”, “PM” = Princess Margaret Cancer Centre, “RD” = Registered Dietitian, Home Care and Other Hospital referrals assume seen by RD (Yes).

In the pre and post groups, 51/140 (36.4%) and 117/117 (100%) were referred to an RD, respectively, considering all patients, *p* < 0.001 (Figure 2). A total of 27.2% of patients were not referred to an RD, and 7.4% were already seeing an RD. Most referrals occurred after the initial OACC visit (94.0%), with 91.7% referred to a PM RD. Among those referred, referral to a PM RD was higher in the post group (100%) compared to the pre group (72.5%). A few patients in the pre group were referred to home care RD (25.5%) or another hospital (2.0%). Of the total referred patients, a slightly higher rate of patients saw an RD in the post group (83.8%) than in the pre group (74.5%). Reasons for not being seen by an RD are shown in Figure 3.

Further stratifying patients into at-risk and malnourished groups, at-risk patients were referred more often in the post group than the pre group (100% vs. 37%, *p* < 0.001). Similarly, for malnourished patients, 100% were referred in the post group versus 84.6% in the pre group (*p* = 0.011) (Supplementary Table S2).

#### 3.2.3. RD Nutritional Risk Judgement

In the pre group, 50.0%, 45.8%, and 4.2% were deemed high, moderate, and low nutritional risk by the RD, respectively, while the post group had 60.2%, 34.7%, and 5.1% deemed high, moderate, and low nutritional risk by the RD, respectively (*p* = 0.59) (Supplementary Table S3).

### 3.3. Objective 2: Malnutrition Assessment and Management

#### 3.3.1. Key Symptoms of Malnutrition, Nutritional Prescription with RD

Key symptoms of malnutrition were reported by the RD in nearly all patients in both the pre and post groups, with 95.8% versus 96.9% (*p* = 0.78). There was a nutritional prescription (100%) in all patients who saw an RD across both groups (Supplementary Table S4).

#### 3.3.2. Nutritional Education by RD vs. OACC MD/RN

All patients (100%) who saw an RD received nutritional education from the RD both pre- and post-intervention. However, the nutritional education provided by the OACC MD/RN increased significantly from 43/140 (30.7%) in the pre group to 60/117 (51.3%) in the post group (*p* < 0.001, Supplementary Table S4). For patients deemed at risk by the OACC MD, 38/119 (31.9%) in the pre group received nutritional education by the OACC MD/RN compared to 34/117 (54.0%), *p* = 0.003. Similarly, for malnourished patients, 5/21 (23.8%) in the pre group received nutrition education by the OACC MD/RN, compared to 24/41 (58.5%) in the post group (*p* = 0.009, Supplementary Figure S3).

#### 3.3.3. Referrals to Other Healthcare Professionals by RD

Across all specialties, the post group had more referrals than the pre group, highlighting the improved access to interdisciplinary care facilitated by the integration of an RD into the patient care pathway. Referrals to speech-language pathology (SLP), social work (SW), dentistry, and other physicians were exclusively observed post-intervention, with seven referred to SW, six patients referred to SLP, six to dentistry, and one to other physician specialists (the oncologist) (Supplementary Table S5). The largest difference was seen in referrals back to the referring physician (OACC geriatrician), where 1 patient was referred in the pre group compared to 23 post intervention.

#### 3.3.4. Follow-Ups with RD

Among patients that saw an RD, the mean number of follow-ups in the pre group was 1.2 (range 0–10), which was slightly lower compared to in the post group, 1.7 (range 0–14); however, the difference was not statistically significant (*p* = 0.44) (Supplementary Table S4).

### 3.4. Objective 3: Usefulness of the CNST

#### Screening Implementation and Comparison of CNST, OACC MD, and RD Nutritional Risk Judgements

The CNST was recorded in 113 (96.6%) patients in the post population. Within this population, 71 (62.8%) screened positive and 42 (37.2%) screened negative. Of the four patients that did not have a CNST recorded, three cases occurred in the first 5 months after introducing the CNST. From July 2023 onwards, the CNST was recorded in all patients seen, demonstrating rapid uptake and implementation of the CNST in the clinic (Supplementary Table S6).

Among CNST-positive patients, 47.9% were determined to be malnourished, 52.1% at risk, and none as normal by the OACC geriatric clinical nurse specialist and geriatrician. Among CNST negative patients, only 16.7% were deemed malnourished, 57.1% at risk, and 26.2% as normal (*p* < 0.001). While all CNST-positive patients were referred to an RD, 81.7% of these patients saw an RD. Reasons for not being seen are shown in Table 5. Based on the RD nutritional risk judgement, 67.2%, 31%, and 1.7% of the CNST-positive patients were classified as high, moderate, and low nutrition risk, compared to 44.4%, 44.4%, and 11.1% of CNST-negative patients (*p* = 0.03, Table 5). Among CNST-negative patients with clinically significant weight loss, the majority were classified by the RD as high risk (50.0%), moderate risk (37.5%), and low risk (12.5%) (*p* = 0.41). For OACC MD-identified malnourished patients, 68.3% were classified as high risk, 26.8% as moderate, and 2.9% as low risk by the RD (Supplementary Table S7).

## 4. Discussion

We evaluated the impact of integrating an RD into an academic geriatric oncology clinic on nutritional outcomes and management of malnutrition in older adult cancer patients. By comparing the pre- and post-RD groups, our findings demonstrated that prior weight loss was very common in older adults with cancer, yet similar weight change was observed between the pre and post groups over three months. Malnutrition was identified by the OACC MD more often in the post group compared to the pre group. Timeliness of assessment showed no significant difference in the time from referral to first contact attempt, number of contact attempts, and time from referral to initial RD assessment for the pre and post groups. There was a significant improvement in the number of patients referred to an RD in the post group compared to the pre group, particularly those with higher nutritional risk. Nutritional education was provided by the OACC MD/RN significantly more in the post group than in the pre group. This increase suggests improved interdisciplinary collaboration, reinforcing the importance of nutrition in patient care and enhancing team-based management of malnutrition. There was an increase in the number of referrals to other healthcare professionals in the post period, further demonstrating a greater collaboration in patient care. We also examined the uptake of the CNST, which was rapid and feasible. Furthermore, the CNST identified a larger proportion of patients who had a higher nutritional risk deemed by the RD.

To our knowledge, this is the first study to describe and assess the impact of integrating an RD into a geriatric oncology clinic. The existing literature primarily consists of RCTs examining nutritional interventions over shorter periods. These RCTs have shown increases in weight or less weight loss among patients seen by an RD compared to usual care. For example, in a study by Isenring et al., the intervention group had a mean weight change of −0.4 kg, while the usual care group lost −4.7 kg over 12 weeks [11]. In our study, both groups experienced a similar mean weight loss of about 1.2–1.4 kg over 3 months. Importantly, not all RCTs have been positive. A systematic review by Richards et al. evaluated the impact of specific nutrition interventions (nutrition counselling, oral nutrition supplement, or combination of both) in oncology patients undergoing active cancer treatment and found that only 7 of 15 studies demonstrated less weight loss for the nutritional intervention group [27]. Preventing further weight loss is the gold standard for cancer patients who are already at an increased nutritional risk, as it can help reduce the likelihood of developing cachexia and sarcopenia. Possible explanations for the lack of a significant difference in weight loss in our study include the small sample size, limited follow-up, and the inability to control for multiple factors such as cancer site, stage, and treatment. An additional advantage of having an RD integrated within a multidisciplinary team is the greater collaboration, as seen with multiple coordinated referrals to other health care professionals after the patient saw the RD.

A strength of our study is the insight it provides into nutritional resources required to meet the needs of older adults with cancer. According to Cancer Care Ontario, it is recommended that patients should receive an initial consult within 14 days of referral; however, the median wait times were shorter with 14 days and 21 days for the pre and post groups, respectively [28]. Notably, the median wait time for patients deemed at high nutritional risk by the RD was 14 days for both groups, which aligns with the UHN Standards of Care and reflects the prioritization of RD resources. Thus, for a clinic seeing 10–11 new patients a week, our findings emphasize the need for additional RD resources within the clinic setting.

Furthermore, the CNST demonstrated its utility in identifying patients at higher nutritional risk, aligning with RD assessments. Although the literature on the CNST in geriatric oncology is limited, our findings support its rapid implementation and feasibility in clinical practice [18]. The CNST effectively identified a larger proportion of patients deemed to be a high nutritional risk by the RD. However, when the CNST was negative but the patient had clinically significant weight loss, a substantial proportion of these patients were deemed to be high nutritional risk when assessed by the dietitian. This suggests that one should rely on both the CNST result and whether the patient had clinically significant weight loss when considering referring to a dietitian. The findings suggest that the CNST serves as a quick and useful screening tool for identifying malnutrition risk in a geriatric oncology clinic. Further study of the CNST in ambulatory oncology clinics is warranted.

The clinical implications from this study include continued support and expansion of the RD role in our clinic, as well as the continued use of the CNST. Additionally, our team has a greater awareness of educating families and patients about avoiding further weight loss and setting realistic goals for weight gain. Although making specific recommendations to oncologists and patients is beyond the scope of our study, more detailed information can be found in these two review articles [29,30], the ESPEN Clinical Nutrition in Cancer Guideline [23], the Nourish website [22], and the Older Adults with Cancer learning module [31].

A key strength of this study is the real-world setting, which offers practical insights into patient care and clinical workflow. The moderate sample size and comparison between pre- and post-intervention groups also strengthened the validity of our findings. However, limitations exist. The retrospective design limits the ability to establish causality, as observed improvements may also reflect changes in healthcare practices, patient behavior, or treatments. The potential Hawthorne effect, where behaviors change due to the RD’s presence, may have influenced referral rates and nutrition education. Additionally, limited assessment data in the pre period, such as missing assessments by Home Care or Other Hospital RDs, makes it challenging to fully distinguish the RD’s impact. Another limitation is that, while frailty was frequently discussed in medical oncology notes, nutrition was not explicitly mentioned as a distinct component. This lack of specificity makes it difficult to determine whether nutritional concerns were considered as part of frailty assessments and how much they impacted the final treatment decision. Finally, data on other factors, such as adherence to nutrition plans, treatment-related outcomes (e.g., toxicity), patient perspectives, or quality of life, were not collected; such data could have provided additional insights.

## 5. Conclusions

In summary, integrating an RD into the OACC improved malnutrition screening/identification and management, emphasizing the importance of addressing nutrition in cancer care. Key limitations of this study included a retrospective pre–post design from a single center and a modest sample size. Our findings support the inclusion of RDs in multidisciplinary teams to optimize patient care, especially for older adults with cancer facing unique nutritional challenges.

Future research should include longitudinal, prospective studies or RCTs to better assess the long-term impact of RD interventions on nutritional outcomes, quality of life, and clinical recovery in older cancer patients. Incorporating patient-reported outcomes, such as nutritional satisfaction and quality of life, would provide a more comprehensive evaluation.

## Figures and Tables

**Table 1:** Outcome measures, definition, and source for each objective.

Objective	Outcome Measure	Outcome Measure Definition Pre OACC RD	Outcome Measure Definition Post OACC RD	Source of Outcome Measure
(1) To assess nutrition-related patient outcomes prior to and after the introduction of a dietitian in the OACC	Weight Characteristics	Body Mass Index measured by OACC MD/RN	Body Mass Index measured by OACC MD/RN	OACC consult note in Epic (electronic health record)
		Initial RD Assessment Weight	Initial RD Assessment Weight	Extracted from RD’s notes on Epic or RD’s notes
		Prior weight loss at OACC consult	Prior weight loss at OACC consult	Self-reported by patient and taken from initial OACC consult note in Epic
		Clinically significant weight loss based on the UHN Clinical Nutrition Prioritization matrix defined as unintentional weight loss meeting any of the following criteria: greater than 2% within 1 week, 5% within 1 month, 7.5% within 3 months, or 10% within 6 months [26]	Clinically significant weight loss based on the UHN Clinical Nutrition Prioritization matrix defined as unintentional weight loss meeting any of the following criteria: greater than 2% within 1 week, 5% within 1 month, 7.5% within 3 months, or 10% within 6 months [26]	Calculate weight % change from self-reported weight loss by patient and baseline weight in initial OACC consult note. Determine if weight % change in patient reported time frame meets UHN Clinical Prioritization Matrix
		Weight change between initial OACC consult and 3 ± 1 months after initial OACC consult	Weight change between initial OACC consult and 3 ± 1 months after initial OACC consult	Initial weight and follow up weight taken from Epic or RD excel spreadsheet notes.
			Positive CNST	OACC initial consult note in Epic
	Treatment Impact	Was nutrition explicitly mentioned in the treatment recommendation from OACC? Yes/No	Was nutrition explicitly mentioned in the treatment recommendation from OACC? Yes/No	Found in OACC consult note under cancer treatment decision making recommendation
		Was nutrition explicitly mentioned in final treatment plan from oncology? Yes/No	Was nutrition explicitly mentioned in final treatment plan from oncology? Yes/No	Found in Epic notes from oncologist
(2) To assess whether malnutrition was adequately assessed and managed when identified	Malnutrition Screening/Identification	Time between referral request and first contact attempt from RD	Time between referral request and first contact attempt from RD	Found in RD’s notes by comparing date of referral to first contact attempt
		Number of contact attempts by RD	Number of contact attempts by RD	Found in RD’s notes
		Time between referral request and initial assessment from RD	Time between referral request and initial assessment from RD	Found in Epic by comparing date of referral request and date of initial assessment by the RD
		Referral to RD: Yes/No If yes, which dietitian, PM or Home Care or Other Hospital	Referral to RD: Yes/No If yes, which dietitian, PM or Home Care or Other Hospital	Found in initial OACC consult and first follow up notes and referral request in Epic
		Referral to RD During Follow up OACC Visit Yes/No	Referral to RD During Follow up OACC Visit Yes/No	Found in follow up OACC note and referral request in Epic
		Seen by RD: Yes/No	Seen by RD: Yes/No	Found in RD’s initial assessment notes in Epic
		Reason not seen by RD: patient declined to see RD/Unreachable (RD contact attempt but no answer)/Unwell or Deceased/Other	Reason not seen by RD: patient declined to see RD//Unreachable (RD contact attempt but no answer)/Unwell or Deceased/Other	Found in Epic or RD’s notes
		Standard of Care Clinical Nutrition Prioritization Matrix –Assessment of Nutritional Risk: High/Moderate/Low (Supplementary Figure S1)	Standard of Care Clinical Nutrition Prioritization Matrix—Assessment of Nutritional Risk: High/Moderate/Low (Supplementary Figure S1)	Determined by the RD initial assessment and follow ups in Epic or RD Notes
	Malnutrition Assessment and Management	Key symptoms of malnutrition: appetite, constipation, muscle weakness Yes/No	Key symptoms of malnutrition: appetite, constipation, muscle weakness Yes/No	Under nutrition focused findings in initial assessment by the RD in Epic
		Nutritional prescription: Were recommendations made by RD: dietary (energy/protein/fluids requirements), medications (e.g., modifying bowel routine, correcting electrolyte imbalances), investigations (blood work, x-rays)? Yes/No	Nutritional prescription: Were recommendations made by RD: dietary (energy/protein/fluids requirements), medications (e.g., modifying bowel routine, correcting electrolyte imbalances), investigations (blood work, x-rays)? Yes/No	Found in RD’s notes or in Epic, specifically under nutritional interventions and recommendation
		Nutrition education: Was education on nutrition provided by RD? Yes/No	Nutrition education: Was education on nutrition provided by RD? Yes/No	Found in RD’s notes under nutritional interventions and recommendation
		Nutrition education: Was education on nutrition provided by OACC MD/RN? Yes/No	Nutrition education: Was education on nutrition provided by OACC MD/RN? Yes/No	Found in OACC consult note
		Referral to other healthcare professionals: speech language pathologist, social worker, dentistry, OACC MD physician, other (physician specialties) Yes/No	Referral to other healthcare professionals: speech language pathologist, social worker, dentistry, OACC MD physician, other (physician specialties) Yes/No	Found in RD’s spreadsheet notes or in the OACC initial consult or first initial assessment by RD in Epic
		Number of follow up with RD within 3 ± 1 months after initial OACC consult	Number of follow up with RD within 3 ± 1 months after initial OACC consult	Extracted from RD’s notes in Epic
(3) To examine the usefulness of the CNST in identifying malnutrition risk	Nutritional Risk Determined by RD vs. CNST vs. OACC MD		Comparison of high/moderate/low nutritional risk determined by RD based on standard of care criteria, compared to CNST positive/negative, and OACC MD normal/at risk/malnourished nutritional risk diagnosis (clinical judgement). Additional comparison of CNST negative patients with clinically significant weight loss and their corresponding RD nutrition risk.	Data found in Epic and in RD’s notes

Abbreviations: OACC, Older Adults with Cancer Clinic, RD, Registered Dietitian, MD, Doctor of Medicine, RN, Registered Nurse, UHN, University Health Network, CNST, Canadian Nutrition Screening Tool.

**Table 2:** Baseline characteristics of total (N = 257), pre (N = 140), and post (N = 117) patients.

Characteristic	Total Mean (SD) or N (%)	Pre Mean (SD) or N (%)	Post Mean (SD) or N (%)
Age, y	80.7 (6.4)	80.2 (6.4)	81.4 (6.5)
Female Sex	138 (53.7%)	68 (48.6%)	70 (59.8%)
VES-13 Score ≥ 3 (Vulnerable)	203 (79.0%)	109 (77.9%)	94 (80.3%)
Disease Site	Head and Neck 61 (23.7%) GI 53 (20.6%) Gyne 41 (16.0%) Lymphoma 27 (10.5%) GU 20 (7.8%) Thoracic 20 (7.8%)	Head and Neck 41 (29.3%) GI 34 (24.3%) Gyne 19 (13.6%) Lymphoma 14 (10.0%) GU 11 (7.9%) Thoracic 8 (5.7%)	Gyne 22 (18.8%) Head and Neck 20 (17.1%) GI 19 (16.2%) Lymphoma 13 (11.1%) Thoracic 12 (10.3%) Breast 10 (8.5%)
Treatment Intent			
Palliative	99 (38.5%)	55 (39.3%)	44 (37.6%)
Curative	127 (49.4%)	74 (52.9%)	53 (45.3%)
Unknown	31 (12.1%)	11 (7.9%)	11 (9.4%)
Reason for Referral			
Pre Treatment	198 (77.0%)	112 (80.0%)	86 (73.5%)
Active Treatment ^a^	39 (15.2%)	15 (10.7%)	24 (20.5%)
Other	20 (7.8%)	13 (9.3%)	7 (6.0%%)
Current Treatment ^a^			
Yes	108 (42.0%)	51 (36.4%)	57 (48.7%)
Current Treatment Modality			
Chemotherapy	30	13	17
Multimodal	16	8	8
Surveillance	16	8	8
Surgery	15	9	6
Radiation	15	5	10
Hormone Therapy	9	4	5
Immunotherapy	5	1	4
Other	5	2	3
Targeted Therapy	3	2	1
Proposed Treatment Modality			
Surgery	75	47	28
Chemotherapy	52	30	22
Multimodal ^b^	46	23	23
Radiation	19	10	9
Immunotherapy	6	4	2
Hormone Therapy	4	2	2
Other	4	1	3
Targeted	3	1	2
Surveillance/Supportive Care	0	0	0
GERIATRIC DOMAINS ^c^			
Comorbidity			
High	39 (15.2)	20 (14.3)	19 (16.2)
Moderate	82 (31.9)	42 (30.0)	40 (34.2)
Low	136 (52.9)	78 (55.7)	58 (49.6)
Functional Status -IADLs			
Independent	94 (36.6)	54 (38.6)	40 (34.2)
Dependent in 1 + IADLs	163 (63.4)	86 (61.4)	77 (65.8)
Functional Status—Physical Performance			
Normal	59 (23.0)	32 (22.9)	27 (23.1)
Decreased	195 (75.9)	105 (75.0)	90 (76.9)
Deferred	3 (1.2)	3 (2.1)	0 (0.0)
Falls Risk			
Increased	168 (65.4)	91 (65.0)	77 (65.8)
Not Increased	87 (33.9)	47 (33.6)	40 (34.2)
Deferred	2 (0.8)	2 (1.4)	0 (0.0)
Medication Optimization			
Potential for Optimization	176 (68.5)	87 (62.1)	89 (76.1)
No Issues	81 (31.5)	53 (37.9)	28 (23.9)
Social Support			
Good	171 (66.5)	98 (70.0)	73 (62.4)
Vulnerable	84 (32.7)	41 (29.3)	43 (36.8)
None	2 (0.8)	1 (0.7)	1 (0.9)
Mood			
Normal	183 (71.2)	106 (75.7)	77 (65.8)
Depressed	60 (23.3)	26 (18.6)	34 (29.1)
Unable to Assess Fully	14 (5.4)	8 (5.7)	6 (5.1)
Cognition			
Normal	127 (49.4)	68 (48.6)	59 (50.4)
Abnormal	68 (26.5)	40 (28.6)	28 (23.9)
Borderline/Req Further Testing	62 (24.1)	32 (22.9)	30 (25.6)
Nutrition			
Malnourished	62 (24.1)	21 (15.0)	41 (35.0)
At Risk	182 (70.8)	119 (85.0)	63 (53.8)
Normal	13 (5.1)	0 (0.0)	13 (11.1)

Abbreviations: VES-13, Vulnerable Elders Survey-13, GI, Gastrointestinal, GU, Genitourinary, IADLs, Instrumented Activities of Daily Living, Gyne, Gynecological, Req, Requires, ^a^ Treatment ongoing or within the last 30 days. ^b^ Any combination (2 or more) active treatment modalities. ^c^ Numbers in parentheses represent percentages.

**Table 3:** Weight characteristics at initial OACC visit, initial RD assessment, at RD assessment for the total, pre, and post patient population.

Characteristic	Total Mean (SD) or N (%)	Pre Mean (SD) or N (%)	Post Mean (SD) or N (%)	Between Groups, *p* Value
Body Mass Index (kg/m^2^)	24.8 (5.7)	25.1 (6.0)	24.5 (5.3)	0.39
Initial Weight at RD Assessment (kg)	62.2 (14.7)	67.6 (14.3)	62.1 (14.7)	0.10
Prior Weight Loss				
Yes	217 (84.4)	114 (81.4)	103 (88.0)	
Yes, quantifiable	191 (88.0)	102 (89.5)	89 (86.4)	
Weight Loss (kg) ^a^	6.8 (4.5–10.0)	6.8 (4.5–9.8)	6.0 (3.2–9.8)	0.19
Time of Weight Loss (months)	6.3 (2.9)	6.5 (3.2)	6.0 (2.5)	
Clinically Significant Weight Loss ^b^	80 (41.9)	40 (40.2)	39 (43.8)	0.61
Weight Change (kg)	−1.2 (4.6)	−1.4 (5.3)	−1.2 (4.0)	0.77
Time period of Weight Change (days) ^c^	90.8 (14.9)	90.6 (15.8)	90.9 (14.4)	0.90
Reported Weight Change	158 (61.5)	70 (50.0)	88 (75.2)	
Weight Loss of ≥ 5 kg	26 (16.5)	13 (18.6)	13 (14.8)	0.52
Missing Weight	99 (38.5)	70 (50.0)	29 (24.8)	<0.001
Reason for Missing Weight				
Not Seen at Hospital	45 (45.5)	31 (44.3)	14 (48.3)	
Seen but Not Recorded	46 (46.5)	38 (54.3)	8 (27.6)	
No Initial Weight	8 (8.1)	1 (1.4)	7 (24.1)	

Abbreviations: Kg = kilogram, OACC, Older Adults with Cancer Clinic, RD, Registered Dietitian, SD, Standard Deviation, ^a^ results report median (interquartile range), ^b^ Clinically significant weight loss defined as unintentional weight loss meeting any of the following criteria: greater than 2% within 1 week, 5% within 1 month, 7.5% within 3 months, or 10% within 6 months, ^c^ weight measured within a range of 60 to 120 days after initial OACC visit, accounting for a 1-month margin before or after the 3-month target.

**Table 4:** Time between referral and initial assessment with registered dietitian and referral to first contact attempt in total (N = 122), pre (N = 24), post (N = 98) patients.

Characteristic	Total	Pre	Post	High Risk Pre	High Risk Post	Between Groups, *p* Value ^a^
Referral to First Contact Attempt (Days)						
Mean (SD)	16.9 (12.6)	19.9 (18.7)	16.2 (10.7)	18.6 (17.7)	14.3 (10.4)	0.20
Median (IQR)	14 (9–23)	14 (7–26.25)	14 (10–22)	14 (8–27.8)	13 (7–19.5)	0.97
Range	0–73	0–67	2–73	0–62	2–73	
Number of Contact Attempts Mean (SD)	1.16 (0.46)	1 (0)	1.20 (0.51)	1 (0)	1.12 (0.50)	0.06
Referral to Initial Assessment (Days)						
Mean (SD)	25.5 (22.7)	20.4 (18.4)	26.7 (23.6)	19.6 (17.1)	21.8 (24.1)	0.23
Median (IQR)	20 (10–32)	14 (8.5–26.3)	21 (10–34)	14 (9–27.7)	14 (7–23.5)	0.18
Range	2–138	2–67	2–138	2–63	2–138	

Abbreviations: SD, Standard Deviation, IQR, Interquartile Range, ^a^ *p* value comparison between pre and post.

**Table 5:** CNST-positive (N = 71) and CNST-negative (N = 42) vs. OACC nutritional clinical judgement, RD nutritional risk judgement, and seen by RD.

Characteristic	CNST Positive N (%)	CNST Negative N (%)	Between Groups, *p* Value
**Total**	**N = 71**	**N = 42**	
OACC MD Nutritional Clinical Judgement			
Malnourished	34 (47.9)	7 (16.7)	<0.001
At Risk	37 (52.1)	24 (57.1)	
Normal	0 (0.0)	11 (26.2) *	
Seen by RD			
Yes	58 (81.7)	36 (85.7)	
No	13 (18.3)	6 (14.3)	
Reason Not Seen			
Unreachable	6 (46.2)	0 (0.0)	
Unwell/Deceased	2 (15.4)	2 (33.3)	
RD Nutritional Risk Judgement ^a^			
High	39 (67.2)	16 (44.4)	0.03
Moderate	18 (31.0)	16 (44.4)	0.006 ^b^
Low	1 (1.7)	4 (11.1)	
RD Nutritional Risk Judgement with Patients Who Had Clinically Significant Weight Loss but were CNST negative (n = 8)			
High		4 (50.0)	
Moderate		3 (37.5)	
Low		1 (12.5)	

Abbreviations: CNST, Canadian Nutrition Screening Tool, OACC = Older Adults with Cancer Clinic, MD, Doctor of Medicine, RD, Registered Dietitian, * Patient referred during follow up with nutrition concerns after starting treatment (n = 5), or requested to speak with a dietitian (n = 2), or required specific dietary advice (increase protein, food security) (n = 2), or people who had decreased appetite or weight loss that was less than the clinically significant weight loss cut offs (n = 2), ^a^ Totals may not add up to 100% due to rounding, ^b^ *p* value comparison between high and moderate RD nutritional risk judgement in CNST-positive patients. This table only includes patients in the post-RD period after the introduction of the CNST.

## Data Availability

Data are not available due to ethical restrictions from the Quality Improvement board. Specific queries can be sent to the corresponding author.

## Supplementary Materials

The following supporting information can be downloaded at: https://www.mdpi.com/article/10.3390/nu17091591/s1, Figure S1: University Health Network Standard of Care Matrix, Figure S2: Boxplot of the Time (days) from Referral to Initial RD Assessment in Pre and Post Groups, Figure S3: Percentage of At Risk or Malnourished Patients Receiving Nutrition Education in the Pre and Post Population, Table S1: Treatment Impact, Table S2: Comparison of Referral to RD in At Risk and Malnourished Patients Deemed by the OACC MD, Table S3: RD Nutritional Risk Judgement for Total (N = 122), Pre (N = 24), Post (N = 98), Table S4: Key Symptoms of Malnutrition, Nutritional Prescription, Nutrition Education by RD and OACC MD/RN, Number Follow Ups, in Total (N = 122), Pre (N = 24), Post (N = 98) Patients^a^,Table S5: Frequency of Patient Referrals to Different Healthcare Specialists by an RD in the Pre and Post Period, Table S6: Quartile Analysis of CNST Not Recorded (N = 4), Table S7: Comparison of RD Nutritional Risk Judgement Against OACC MD Nutritional Judgement of Malnourished (N = 70), At Risk (N = 41), and Normal (N = 11) Patient Population.

## References

[1] Strohschein F.J., Newton L., Puts M., Jin R., Haase K., Plante A., Loucks A., Kenis C., Fitch M. (2021). Optimizing the Care of Older Canadians with Cancer and their Families: A Statement Articulating the Position and Contribution of Canadian Oncology Nurses. Can. Oncol. Nurs. J..

[2] Blackberry I., Boak J., Rasekaba T., Steer C. (2025). Real-world implementation of geriatric assessment in cancer care among older adults: The role of implementation science frameworks. Curr. Opin. Support. Palliat. Care.

[3] Dale W., Klepin H.D., Williams G.R., Alibhai S.M.H., Bergerot C., Brintzenhofeszoc K., Hopkins J.O., Jhawer M.P., Katheria V., Loh K.P. (2023). Practical Assessment and Management of Vulnerabilities in Older Patients Receiving Systemic Cancer Therapy: ASCO Guideline Update. J. Clin. Oncol..

[4] Wildiers H., Heeren P., Puts M., Topinkova E., Janssen-Heijnen M.L., Extermann M., Falandry C., Artz A., Brain E., Colloca G. (2014). International Society of Geriatric Oncology consensus on geriatric assessment in older patients with cancer. J. Clin. Oncol..

[5] Magnuson A., Loh K.P., Stauffer F., Dale W., Gilmore N., Kadambi S., Klepin H.D., Kyi K., Lowenstein L.M., Phillips T. (2024). Geriatric assessment for the practicing clinician: The why, what, and how. CA Cancer J. Clin..

[6] Mohile S.G., Dale W., Somerfield M.R., Schonberg M.A., Boyd C.M., Burhenn P.S., Canin B., Cohen H.J., Holmes H.M., Hopkins J.O. (2018). Practical Assessment and Management of Vulnerabilities in Older Patients Receiving Chemotherapy: ASCO Guideline for Geriatric Oncology. J. Clin. Oncol..

[7] Kleckner A.S., Magnuson A. (2022). The nutritional needs of older cancer survivors. J. Geriatr. Oncol..

[8] Siwakoti K., Nabell L., McDonald A.M., Williams G.R. (2024). Association of malnutrition with geriatric assessment impairments and health-related quality of life among older adults with head and neck cancers. J. Geriatr. Oncol..

[9] Williams G.R., Al-Obaidi M., Dai C., Mir N., Challa S.A., Daniel M., Patel H., Barlow B., Young-Smith C., Gbolahan O. (2020). Association of malnutrition with geriatric assessment impairments and health-related quality of life among older adults with gastrointestinal malignancies. Cancer.

[10] Vitaloni M., Caccialanza R., Ravasco P., Carrato A., Kapala A., de van der Schueren M., Constantinides D., Backman E., Chuter D., Santangelo C. (2022). The impact of nutrition on the lives of patients with digestive cancers: A position paper. Support. Care Cancer.

[11] Isenring E.A., Capra S., Bauer J.D. (2004). Nutrition intervention is beneficial in oncology outpatients receiving radiotherapy to the gastrointestinal or head and neck area. Br. J. Cancer.

[12] Dai W., Wang S.A., Wang K., Chen C., Wang J., Chen X., Yan J. (2022). Impact of Nutrition Counseling in Head and Neck Cancer Sufferers Undergoing Antineoplastic Therapy: A Randomized Controlled Pilot Study. Curr. Oncol..

[13] Ravasco P., Monteiro-Grillo I., Camilo M. (2012). Individualized nutrition intervention is of major benefit to colorectal cancer patients: Long-term follow-up of a randomized controlled trial of nutritional therapy. Am. J. Clin. Nutr..

[14] Sullivan E.S., Rice N., Kingston E., Kelly A., Reynolds J.V., Feighan J., Power D.G., Ryan A.M. (2021). A national survey of oncology survivors examining nutrition attitudes, problems and behaviours, and access to dietetic care throughout the cancer journey. Clin. Nutr. ESPEN.

[15] Trujillo E.B., Claghorn K., Dixon S.W., Hill E.B., Braun A., Lipinski E., Platek M.E., Vergo M.T., Spees C. (2019). Inadequate Nutrition Coverage in Outpatient Cancer Centers: Results of a National Survey. J. Oncol..

[16] Roeland E.J., Dunne R.F. (2021). The Impact of Early Referrals to Dietitians for Patients With Esophagogastric Cancer. J. Natl. Compr. Cancer Netw..

[17] Abu Helal A., Chon J., Timilshina N., Berger A., Romanovsky L., Jin R., Monginot S., Alibhai S.M.H. (2022). Quality of care of consultations from the geriatric oncology clinic: “Are we addressing the needs of patients?”. J. Geriatr. Oncol..

[18] Laporte M., Keller H.H., Payette H., Allard J.P., Duerksen D.R., Bernier P., Jeejeebhoy K., Gramlich L., Davidson B., Vesnaver E. (2015). Validity and reliability of the new Canadian Nutrition Screening Tool in the ‘real-world’ hospital setting. Eur. J. Clin. Nutr..

[19] Mislang A.R., Di Donato S., Hubbard J., Krishna L., Mottino G., Bozzetti F., Biganzoli L. (2018). Nutritional management of older adults with gastrointestinal cancers: An International Society of Geriatric Oncology (SIOG) review paper. J. Geriatr. Oncol..

[20] Canadian Malnutrition Task Force (2025). Research–Nutrition Care in Canada [Internet].

[21] Taylor L.M., Eslamparast T., Farhat K., Kroeker K., Halloran B., Shommu N., Kumar A., Fitzgerald Q., Gramlich L., Abraldes J.G. (2021). Using Patient Completed Screening Tools to Predict Risk of Malnutrition in Patients With Inflammatory Bowel Disease. Crohns Colitis 360.

[22] Canadian Taskforce on Anthropometric Measures (CTAN) (2025). Nourish: Nourishing Canada’s Aging Population [Internet].

[23] Muscaritoli M., Arends J., Bachmann P., Baracos V., Barthelemy N., Bertz H., Bozzetti F., Hütterer E., Isenring E., Kaasa S. (2021). ESPEN practical guideline: Clinical Nutrition in cancer. Clin. Nutr..

[24] Almugbel F.A., Timilshina N., AlQurini N., Loucks A., Jin R., Berger A., Romanovsky L., Puts M., Alibhai S.M.H. (2021). Role of the vulnerable elders survey-13 screening tool in predicting treatment plan modification for older adults with cancer. J. Geriatr. Oncol..

[25] Alibhai S.M.H., Jin R., Loucks A., Yokom D.W., Watt S., Puts M., Timilshina N., Berger A. (2018). Beyond the black box of geriatric assessment: Understanding enhancements to care by the geriatric oncology clinic. J. Geriatr. Oncol..

[26] University Health Network (2019). Standards of Care: Clinical Nutrition Prioritization Matrix.

[27] Richards J., Arensberg M.B., Thomas S., Kerr K.W., Hegazi R., Bastasch M. (2020). Impact of Early Incorporation of Nutrition Interventions as a Component of Cancer Therapy in Adults: A Review. Nutrients.

[28] Cancer Care Ontario (2024). Appendix 1-45: CCO DBK, version 2.

[29] Soares C.H., Beuren A.G., Friedrich H.J., Gabrielli C.P., Stefani G.P., Steemburgo T. (2024). The Importance of Nutrition in Cancer Care: A Narrative Review. Curr. Nutr. Rep..

[30] Gray A., Dang B.N., Moore T.B., Clemens R., Pressman P. (2020). A review of nutrition and dietary interventions in oncology. SAGE Open Med..

[31] Princess Margaret Cancer Campus (2025). Malnutrition in Cancer Care [Internet].

