# Supplementary material for: Retrospective Analysis of the Impact of a Dietitian and the Canadian Nutrition Screening Tool in a Geriatric Oncology Clinic
Source: Nutrients. 2025 May 6;17(9):1591. doi: 10.3390/nu17091591 (PMC12073638; doi:10.3390/nu17091591)
Supplement: Supplementary file 1 [file nutrients-17-01591-s001.zip › nutrients-3592527-supplementary.pdf]

Supplemental Tables and Figures

Oncology (Inpatient and Outpatient)

As per the Cancer Care Ontario Psychosocial Oncology Wait Time Expert Panel, ambulatory patients at Princess Margaret Cancer Center will receive a first consult within 2 weeks (14 consecutive days) from the date the referral is received. This standard may not apply to Priority 3 (low nutrition risk) patients.

**Priority 1 (High Risk)**	**Priority 2 (Moderate Risk)**	**Priority 3 (Low Risk)**
• Significant unintentional weight loss (i.e. greater than 2% in 1 week; greater than 5% in 1 month; greater than 7.5% in 3 months; greater than 10% in 6 months)<br>• BMI less than 18.5 undergoing active treatment<br>• Nutrition impact symptoms resulting in significantly decreased intake and/or increased risk of dehydration x 2-3 days<br>• Adverse Events (impacting nutrition status) grade 3 and 4 (NCI CTCAE v5.0)<br>• Presence of pressure ulcers (Braden scale 1 to 4)<br>• Newly inserted gastrostomy or jejunostomy feeding tube for nutrition support therapy<br>• Intolerance to enteral nutrition, such as nausea, vomiting, bloating, diarrhea after feeds<br>• High-risk of refeeding syndrome (poor oral intake for 7 or more days)<br>• NPO/clear fluids x 5 days<br>• Requires new therapeutic diet education or patient on therapeutic diets plus significant weight loss (examples: bowel narrowing/obstruction, post GI surgery, renal)<br>• Textured modified diets for dysphagia	• Anticipated nutrition impact symptoms that will significantly reduce intake<br>• Use of alternate/ complementary therapy, i.e. mega-dosing of vitamins/minerals<br>• A secondary diagnosis/comorbidity with nutrition implications; examples:<br>○ diabetes<br>○ renal failure<br>○ Crohn's disease<br>○ bariatric surgery or gastric bypass<br>• Immunosuppression requiring food safety education<br>• BMI less than 18.5 *not* undergoing active treatment<br>• Existing enteral feeds with adequate tolerance and stable weight status<br>• Difficulty weaning off feeds to resume oral diet<br>• Nutrition impact symptoms resulting in less than 75% of usual intake	• Understanding and application of nutrition and cancer scientific literature<br>• Nutrition therapy to reduce risk of recurrence/secondary cancers<br>• Nutrition education to maintain quality of life<br>• Nutritional therapy for purposes of weight management referred to community/ other ambulatory care registered dietitian such as Family Health Teams.

Supplementary Figure 1 University Health Network Standard of Care Matrix [26]

Supplementary Table 1. Treatment Impact				
**Characteristic**	Total N (%)	Pre N (%)	Post N (%)	Between groups, P value
**Nutrition Mentioned in OACC Recommendation**				
Yes	34 (13.2)	8 (5.7)	24 (22.2)	< 0.001
No	186 (72.4)	108 (77.1)	78 (66.7)	
N/A	37 (14.4)	13 (9.3)	24 (20.5)	
**Nutrition Mentioned in Final Treatment Plan by Oncologist**				
Yes	3 (1.2)	2 (1.4)	1 (0.9)	0.93
No	72 (28.0)	40 (28.6)	32 (27.4)	
N/A	182 (70.8)	98 (70.0)	32 (71.8)	

Abbreviations:

OACC, Older Adults with Cancer Clinic

N/A, Not Applicable

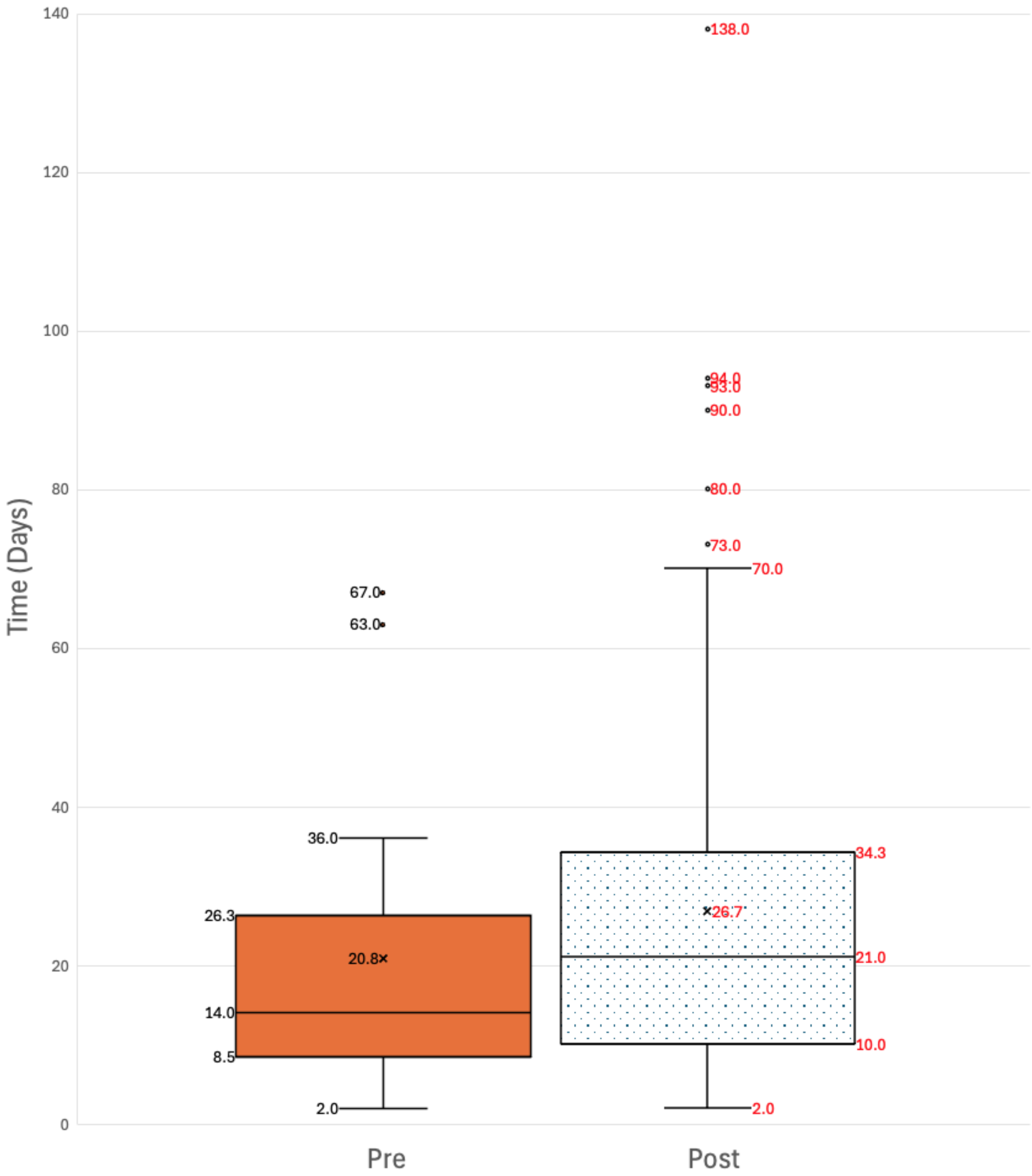
Time (Days)
140
120
100
80
60
40
20
0
Pre
Post
138.0
94.0
93.0
90.0
80.0
73.0
70.0
67.0
63.0
36.0
34.3
26.3
26.7
20.8
21.0
14.0
8.5
10.0
2.0
2.0

Supplementary Figure 2. Boxplot of the Time (days) from Referral to Initial RD Assessment in Pre and Post Groups

Supplementary Table 2. Comparison of Referral to RD in At Risk and Malnourished Patients Deemed by the OACC MD

Characteristic	N (Pre)	N (Post)	Total N	Chi Squared p-value
**At Risk Referred**				
Yes	40	63	103	< 0.001
No	68	0	68	
Total	108	63	171	
**Malnourished Referred**				
Yes	11	41	52	0.011
No	2	0	2	
Total	13	41	54	

Abbreviations:

RD, Registered Dietitian

OACC, Older Adults with Cancer Clinic

MD, Doctor of Medicine

Supplementary Table 3. RD Nutritional Risk Judgement for Total (N = 122), Pre (N = 24), Post (N= 98)

RD Nutritional Risk Judgement	Total N (%)	Pre N (%)	Post Post (N)	Between Groups, P value
High	71 (58.2)	12 (50.0)	59 (60.2)	0.60
Moderate	45 (36.9)	11 (45.8)	34 (34.7)	
Low	6 (4.9)	1 (4.2)	5 (5.1)	

Abbreviations:
RD, Registered Dietitian

Supplementary Table 4. Key Symptoms of Malnutrition, Nutritional Prescription, Nutrition Education by RD and OACC MD/RN, Number Follow Ups, in Total (N = 122), Pre (N = 24), Post (N=98) Patients[a]

Characteristic	Total N[b] (%)	Pre N (%)	Post N (%)	Between Groups, P Value
**Key Symptoms**				
Yes	118 (96.7)	23 (95.8)	95 (96.9)	0.78
No	4 (3.3)	1 (4.2)	3 (3.1)	
**Nutritional Prescription**				
Yes	122 (100.0)	24 (100.0)	98 (100.0)	
No	0 (0.0)	0 (0.0)	0 (0.0	
**Nutrition Education by RD**				
Yes	122 (100.0)	24 (100.0)	98 (100.0)	
**Nutrition Education by OACC MD/RN[c]**				
Yes	103 (40.1)	43 (30.7)	60 (51.3)	< 0.001
No	154 (59.9)	97 (69.3)	57 (48.7)	
**Number of Follow Ups, mean (SD)**	1.6 (2.5)	1.3 (2.2)	1.7 (2.6)	0.44

Abbreviations:
OACC, Older Adults with Cancer Clinic
MD, Doctor of Medicine
RN, Registered Nurse
RD, Registered Dietitian

SD, Standard Deviation

[a] N reflects number of patients seen by RD at PM. Patient seen by Home Care and Other Hospital counts were excluded from denominator as there were no further information available

[b] Numbers in table represent absolute counts and % in brackets unless stated otherwise

[c] Patient denominators Total (n = 257), Pre (n= 140), Post (n = 117)

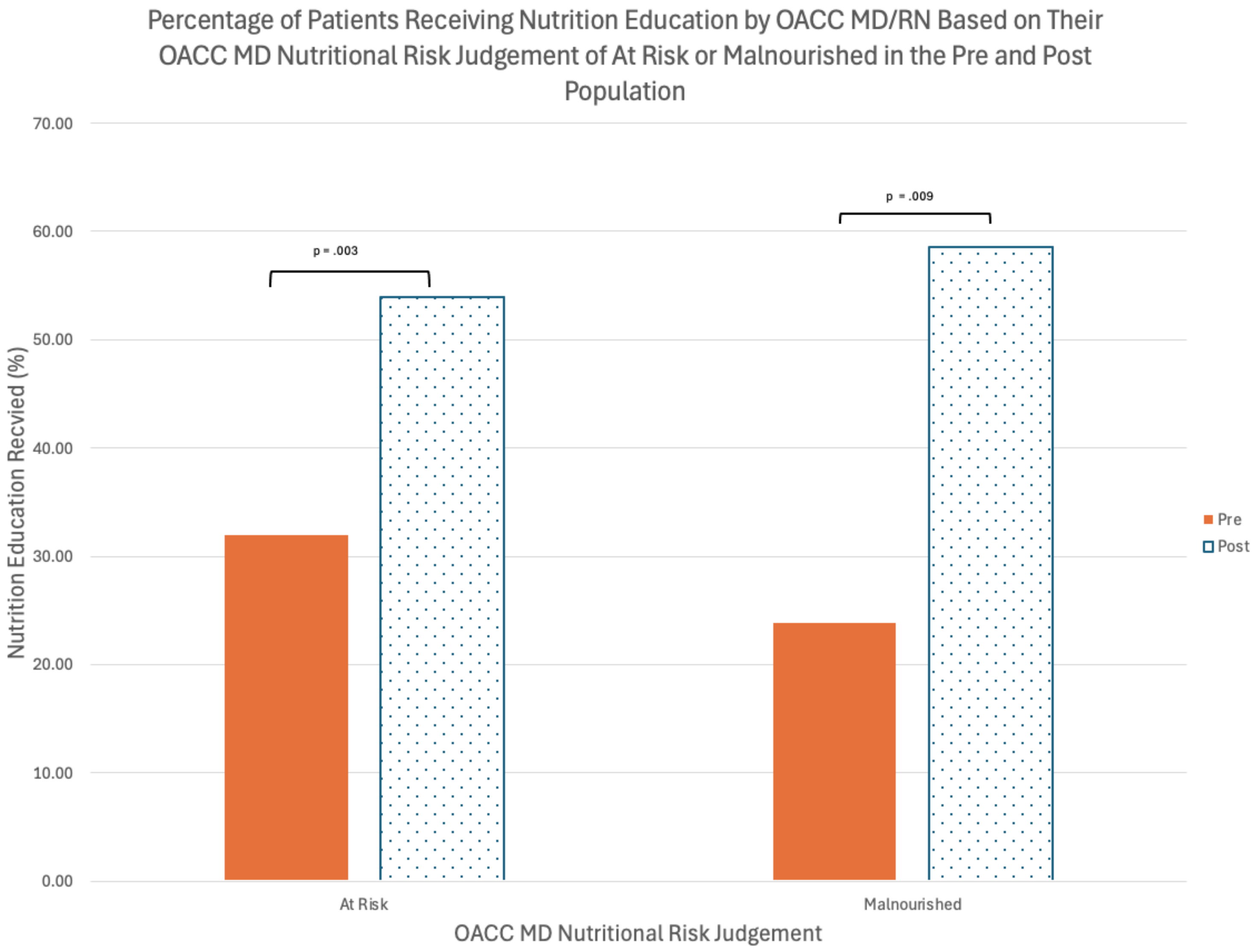

Percentage of Patients Receiving Nutrition Education by OACC MD/RN Based on Their OACC MD Nutritional Risk Judgement of At Risk or Malnourished in the Pre and Post Population
70.00
60.00
50.00
40.00
30.00
20.00
10.00
0.00
Nutrition Education Recvied (%)
p = .003
p = .009
Pre
Post
At Risk
Malnourished
OACC MD Nutritional Risk Judgement

Supplementary Figure 3: Percentage of At Risk or Malnourished Patients Receiving Nutrition Education in the Pre and Post Population. Note: "OACC" = Older Adult with Cancer Clinic", "MD" = Doctor of Medicine, "RN" = Registered Nurse, "RD" = Registered Dietitian

Supplementary Table 5. Frequency of Patient Referrals to Different Healthcare Professionals by an RD in the Pre and Post Period

Healthcare Specialist	Frequency (Pre)	Frequency (Post)
SW	0	7
SLP	0	6
Dentistry	0	6
OACC Geriatrician	1	23
Other Physician	0	1

Abbreviations:
RD, Registered Dietitian
SW, Social Worker
SLP, Speech Language Pathologist
OACC, Older Adults with Cancer Clinic

Supplementary Table 6. Quartile Analysis of CNST Not Recorded (N = 4)

Time Period	Total Patients Seen	N (%)
September 2022 – January 2023	22	3 (13.6)
February 2023 – June 2023	27	1 (3.7)
July 2023 – November 2023	37	0 (0.0)
December 2023 – April 2024	31	0 (0.0)

Abbreviations:
CNST, Canadian Nutrition Screening Tool

Supplementary Table 7. Comparison of RD Nutritional Risk Judgement Against OACC MD Nutritional Judgement of Malnourished (N = 70), At Risk (N = 41), and Normal (N = 11) Patient Population

	Dietitian Judgement		
**OACC MD Nutritional Judgement**	**High N (%)**	**Moderate N (%)**	**Low N (%)**
Malnourished	28 (68.3)	11 (26.8)	2 (2.9)
At Risk	37 (52.9)	31 (44.3)	2 (4.9)
Normal	6 (54.5)	3 (27.3)	2 (18.2)

Abbreviations:

RD, Registered Dietitian

OACC, Older Adults with Cancer Clinic

MD, Doctor of Medicine
